# Park access and mental health among parents and children during the COVID-19 pandemic

**DOI:** 10.1186/s12889-022-13148-2

**Published:** 2022-04-21

**Authors:** Marnie F. Hazlehurst, Sadiya Muqueeth, Kathleen L. Wolf, Cary Simmons, Emily Kroshus, Pooja S. Tandon

**Affiliations:** 1 Department of Epidemiology, University of Washington School of Public Health, Seattle, WA USA; 2 The Trust for Public Land, Washington DC, USA; 3 School of Environmental and Forest Sciences, College of the Environment, University of Washington, Seattle, WA USA; 4 Department of Pediatrics, University of WA, Seattle, WA USA; 5 Seattle Children’s Research Institute, Seattle, WA USA

**Keywords:** Parks, Greenspace, Nature, Mental health, Children, Adolescent, Co-participation, Physical activity, Pandemic

## Abstract

**Background:**

Time spent outdoors and in nature has been associated with numerous benefits to health and well-being. We examined relationships between park access and mental health for children and parents during the COVID-19 pandemic. We also explored associations between park access and co-participation of parent and child in time outdoors, and child and parent physical activity.

**Methods:**

We used data from 1,000 respondents to a nationally representative U.S. survey of parent–child dyads during October–November 2020. Park access was defined as an affirmative response to: “do you have a park that you can safely walk to within 10 min of your home?” Child mental health was operationalized as the Strengths and Difficulties Questionnaire (SDQ) total difficulties score. The Patient Health Questionnaire-4 (PHQ-4) total score assessed parent mental health and the International Physical Activity Questionnaire (IPAQ) assessed parent physical activity. Child physical activity and co-participation in outdoor activity were reported as number of days in the prior week. Linear regression was used to examine relationships between park access and health outcomes in models adjusted for child and parent characteristics and COVID-19 impact.

**Results:**

Our sample included 500 parents of children ages 6–10 years, and 500 parent–child dyads of children ages 11–17 years. Park access was associated with a lower SDQ total score among children (β: -1.26, 95% CI: -2.25, -0.27) and a lower PHQ-4 total score among parents (β: -0.89, 95% CI: -1.39, -0.40). In models stratified by child age, these associations were observed for SDQ scores among adolescents ages 11–17 and for PHQ-4 scores among parents of children ages 6–10 years. Park access was also associated with 0.50 more days/week of co-participation in outdoor time (95% CI: 0.16, 0.84), and higher levels of parent physical activity (β: 1009 MET-min/week, 95% CI: 301, 1717), but not child physical activity (β: 0.31 days/week, 95% CI: -0.03, 0.66).

**Conclusions:**

Park access was associated with better mental health among children and parents, and more parent physical activity and parent–child co-participation in outdooractivity during the COVID-19 pandemic. Access to nearby parks may be an important resource to promote health and well-being, for both individuals and families.

## Introduction

Outdoor time and access to nature have been associated with a range of benefits to health and well-being, including physical activity and mental health [1]. During the ongoing COVID-19 pandemic, outdoor spaces were considered safer than indoors due to lower risk of viral transmission. Pandemic restrictions, such as school closures and cancellation of sports and park program activities, limited opportunities for typical child and parent physical activity. These restrictions further exacerbated existing physical and mental health concerns related to social isolation, sedentary behaviors and less physical activity, including impacts on children and adolescents [2–4].

Prior studies suggest associations between nature contact and improved mental health outcomes across the life course. A recent systematic review of 296 papers synthesized the evidence concerning the relationship between nature contact and children’s health [5]. Among children and adolescents, this literature suggests that higher levels of overall greenness proximate to one’s residential location are associated with better mental health outcomes, particularly for measures of emotional well-being and attention [6, 7]. In adolescents and adults, access to or more time spent in greenspace has been linked to fewer depressive symptoms, even after controlling for socioeconomic factors [8, 9]. While much of the existing literature has focused on overall greenness, public parks specifically may promote physical and mental health, provide more opportunity for direct nature contact, and be a salient exposure from a policy and planning perspective [10].

Several mechanisms have been hypothesized for nature and health relationships, including changes to the physiological stress response such as suppression of sympathetic nervous system activity and healthier diurnal cortisol patterns, increased physical activity, and increased social connections [11–13]. In addition, a socio-ecological framework highlights the potential for relationships between parks and child health to be operating across multiple levels, including direct effects on the individual as well as through family or community pathways.

However, few studies have investigated the influence of park access on parent–child dyads specifically, and little is known as to whether natural environments influence the parent–child relationship. Studies suggest the importance of parent–child attachment and relations for early childhood behavioral and emotional outcomes, including aggression, social stress, and self-esteem [14]. Furthermore, variables related to the quality of family and parental relationships can influence conditions of well-being during adolescence and beyond. For example, self-perception of health status can improve as adolescents perceive a more favorable family climate, including good relations with parents [15]. Outdoor recreation may be a multigenerational activity that is beneficial for health, but literature on this topic is sparse. One study during the COVID-19 pandemic suggests that spending time outdoors may help promote individual well-being and family functioning, particularly if time outdoors involves physical activity and if families spend time physically active outdoors together [16]. Given heightened time demands on many parents to provide child supervision during prolonged school closures, parent and child co-participation in physical activity may be more frequent than they were prior to the pandemic. Most prior studies have focused on either child or adult health outcomes; few studies have explicitly examined how greenspace or nature exposure may facilitate time spent with family, or explored the role that co-participation in outdoor time may play in the relationship between park access and health outcomes. Insight into the potential relationship building opportunities and health promoting role of parks during the heightened stress of the pandemic can inform future policies and programs.

The abrupt and substantial changes in household behavior patterns during the COVID-19 pandemic has provided unique opportunities to investigate how access to parks within residential areas may be related to health behaviors and mental health for families. Using U.S. national survey data, we examined associations between perceived park access and mental health outcomes among children, adolescents and their parents. Measures of child and parent physical activity, including co-participation in outdoor activity, were also investigated.

## Methods

### Study population

This analysis used data from respondents to an online cross-sectional survey conducted by YouGov in the United States during October and November 2020 and was approved by the Seattle Children’s Hospital Institutional Review Board [17]. YouGov interviewed 547 parents of 6–10 year old children, and 535 parent–child dyads with 11–17 year old children. To generate a nationally representative U.S. sample, respondents were then matched down to samples of 500 in each cohort (*n* = 1000 total). The respondents were matched using a three-way sampling frame of age, race, and education for younger children of ages 6–10, and a four-way sampling frame of gender, age, race, and education for adolescent children of ages 11–17 using U.S. census data. Both matched samples were constructed by stratified sampling from the full 2017 American Community Survey (ACS) 1-year sample with selection within strata by weighted sampling with replacements using the person weights on the public use file. Weighting was performed using propensity scores. The full sample of 1000 respondents were also weighted to a sampling frame corresponding to U.S. parents with children 6–17 years of age. Weighting was again performed using propensity scores. The matched cases and the frame were combined and a logistic regression was estimated for inclusion in the frame. The propensity score function included age, gender, race/ethnicity, years of education, and region. The propensity scores were grouped into deciles of the estimated propensity score in the frame and post-stratified according to these deciles. The weights were then post-stratified on a four-way stratification of gender, age (4-categories), race (4-categories), and education (4-categories), to produce the final weight. Sampling weights were used in statistical analysis to reduce bias.

### Park access

A 10-minute walk to a park from one’s home is emerging as a goal and metric for equitable provision of safe park access [18]. This 10-min measure corresponds to an adult walking approximately 0.5 miles; living within this distance to a park has been found to be associated with increased park use and has been used in prior studies [19]. In this study, perceived park access was defined as an affirmative response to the question “Do you have a park that you can safely walk to within 10 min from your home?” Participants could report “yes”, “no”, or “I don’t know”. Participants reporting “no” or “I don’t know” were combined in a single group.

### Strengths and Difficulties Questionnaire (SDQ)

Child internalizing and externalizing behaviors were assessed using the Strengths and Difficulties Questionnaire (SDQ) [20, 21]. Parents reported on behaviors of children ages 6–10 years; behaviors for adolescents 11–17 years were self-reported. Individual items were scored as 0 (“not true”), 1 (“somewhat true”), or 2 (“certainly true”). Items were summed to calculate four difficulties subscales (5 items included in each) and subscales were summed to calculate a total ‘difficulties’ score, internalizing problems, and externalizing problems. The internalizing score included the emotional problems and peer problems subscales and the externalizing score included the conduct problems and hyperactivity subscales. Internalizing, externalizing, and total problems were modeled as continuous scores.

### Parent mental health

Parent mental health was assessed using the four-item Patient Health Questionnaire for Anxiety and Depression (PHQ-4) [22]. Two items are related to anxiety and two questions relate to depression. This questionnaire asks respondents how often they have been bothered by four problems over the prior two weeks: (1) feeling nervous, anxious, or on edge; (2) not being able to stop or control worrying; (3) feeling down, depressed or hopeless; and (4) little interest or pleasure in doing things. Participants selected from the following responses: “not at all”, “several days”, “more than half the days” or “nearly every day”, which were coded as 0, 1, 2, or 3, respectively. The continuous total score, determined by adding together the scores for each of the 4 items, was used as the primary outcome.

### Physical activity

Child physical activity was parent-reported for children ages 6–10 and self-reported for adolescents ages 11–17. Child physical activity was assessed using the following question: “During the past 7 days, on how many days [was your child/were you] physically active for a total of at least 60 min per day? Add up all the time you spent in any kind of physical activity that increased your heart rate and made you breathe hard some of the time”. Participants selected 0 to 7 days and physical activity was modeled as a continuous variable. This 60 min per day cutoff aligns with the current U.S. national recommendation for youth physical activity [23]. Parent physical activity was assessed using the International Physical Activity Questionnaire (IPAQ) short form and calculated as a continuous variable in total MET-minutes per week following standard IPAQ short form scoring procedures [24, 25].

Co-participation in outdoor activity was assessed using the question: “In the past week, on how many days did you go outside with your child for a walk or to play near your home or in a park?” Respondents selected 0 to 7 days, which was modeled as a continuous variable.

### Covariates

Several additional covariates were collected. Parent education was included as a categorical variable with four categories (high school graduate or less, some college or a 2-year degree, 4-year college degree, or a graduate degree). Parent and child race and ethnicity were included as categorical variables (White, Black/African-American, Asian or Asian American, American Indian or Alaska Native, or other and Hispanic or not). Parent born outside of the U.S. or not, was reported as a binary variable. These variables were included as a proxy for unmeasured confounders including factors related to residential segregation. Child gender was included in the model as a binary variable for male or not. Prior child diagnosis of either an anxiety disorder or depression was reported by the parent and included as a binary variable. Prior child diagnosis of Attention Deficit Hyperactivity Disorder (ADHD), autism, a learning disability, or a behavioral problem was also reported. A response of yes to any of these four questions was coded as yes to a prior diagnosis, and no or missing responses for all four questions were considered no prior diagnosis. Urbanicity was reported in five categories: big city, smaller city, suburban area, small town, or rural area.

We also included two potential confounders specifically assessing changes in school status and the economic impact of the pandemic. School status was assessed as three groups: all in-person, hybrid (some days virtual and some days in-person), or all remote/virtual. A shortened version of the COVID-19 Exposure and Family Impact Survey (CEFIS) questionnaire was also used to assess the impact of COVID-19 on the family, including whether family members were employed in essential worker or healthcare worker jobs and whether the family experienced a loss of income due to COVID-19 [26].

### Statistical analysis

Analyses were conducted in R 4.0 (The R Foundation for Statistical Computing; Vienna, Austria). Descriptive statistics were used to explore the characteristics of respondents who did and did not report having park access within a 10-minute walk. Linear regression with robust standard errors was used to assess relationships between park access and child and parent mental health outcomes, as well as to assess relationships between park access and physical activity and co-participation in outdoor time. Stratified models were used to estimate associations among children ages 6–10 and ages 11–17 separately.

Models of child SDQ outcomes were adjusted for age, gender, parent education, child race/ethnicity, COVID-19 impact scale, school status, prior diagnosis of ADHD or behavioral problems, and prior anxiety or depression diagnosis. Models of parent mental health were adjusted for gender, parent education, race/ethnicity, born outside the US, and COVID-19 impact scale. In sensitivity analyses, we further adjusted analyses of mental health for co-participation in outdoor time or physical activity.

Models of physical activity included adjustment for age, gender, parent education, and COVID-19 impact scale. Child physical activity models were additionally adjusted for school status. Co-participation in outdoor activity and parent physical activity models were additionally adjusted for parent race/ethnicity, whether or not the parent was born outside of the US.

## Results

### Descriptive results

This study surveyed 1,000 parents; 64% of respondents reported access to a park within a safe 10-minute walk (Table 1). In the full sample, children were on average 10.8 years old (SD 3.5). The sample included 53% boys. Many families reported impacts of the COVID-19 pandemic on daily life; 51% of children were attending school that was fully remote and only 22% were attending school in-person full time. Additionally, 61% of respondents indicated that someone in the family kept working outside the home (essential personnel) during the pandemic and 19% reported that someone in the family is a healthcare provider/first responder providing direct care.

**Table 1:** Characteristics of the sample overall and by park access

	**Overall**		**Park Access** **(*n* = 632)**		**No Park Access** ^a^ **(*n* = 368)**	
**Child characteristics**						
Age (years), mean (SD)	10.8	(3.5)	10.9	(3.6)	10.6	(3.3)
Boys, n (%)	516	(53)	324	(51)	192	(56)
Race, n (%)						
White	684	(68)	440	(68)	244	(70)
African-American/Black	105	(11)	62	(10)	43	(12)
Asian or Asian American	28	(3)	22	(3)	6	(2)
American Indian or Alaska Native	21	(2)	14	(2)	7	(2)
Other	162	(16)	112	(17)	50	(14)
Ethnicity, n (%)						
Not Hispanic	722	(72)	449	(69)	273	(78)
Hispanic	278	(28)	202	(31)	75	(22)
School status, n (%)						
All in-person	222	(22)	115	(18)	107	(31)
Hybrid	272	(27)	174	(27)	98	(28)
All remote/virtual	505	(51)	363	(56)	143	(41)
**Parent characteristics**						
Gender, n (%)						
Female	554	(55)	346	(53)	208	(60)
Male	446	(45)	305	(47)	141	(40)
Race, n (%) ^b^						
White	714	(71)	455	(70)	259	(74)
African-American/Black	111	(11)	68	(10)	43	(12)
Asian or Asian American	30	(3)	27	(4)	4	(1)
American Indian or Alaska Native	16	(2)	10	(2)	6	(2)
Other	129	(13)	91	(14)	38	(11)
Born outside the US, n (%)						
No	756	(76)	470	(72)	286	(82)
Yes	244	(24)	182	(28)	63	(18)
Education, n (%)						
High school degree or less	376	(38)	253	(39)	124	(35)
Some college or 2-year degree	264	(26)	154	(24)	110	(32)
4-year college degree	225	(23)	146	(22)	79	(23)
Post-graduate degree	134	(13)	99	(15)	36	(10)
Home ownership, n (%)						
Rent	429	(43)	275	(42)	153	(44)
Own	571	(57)	376	(58)	195	(56)
CEFIS, mean (SD) ^c^	2.4	(1.8)	2.5	(2.0)	2.3	(1.5)

^a^18 participants who reported ‘I don’t know’ for park access were included in the no park access category ^b^1 missing parent race (in park access category), 1 missing school status (in no park access category)

^c^CEFIS = COVID-19 Exposure and Family Impact Scale. 2 missing responses for question about whether anyone in the family was a healthcare provider in the (in park access category)

The distribution of mental health outcome measures is shown in Table 2. The average total SDQ score among children was 11.9 (SD 7.1). The average total score was only slightly higher among children ages 6–10 years than in the 11–17 group (12.1 compared to 11.7); externalizing scores were higher in the younger group on average, but average internalizing scores were similar across the two age groups. Among parents, the average total PHQ-4 score was 3.1 (SD 3.3); 15% of participants had scores considered moderate to severe (6 or higher).

**Table 2:** Parent and child physical activity and mental health outcomes

	**Overall** **(*n* = 1000)**		**Ages 6–10 years** **(*n* = 500)**		**Ages 11–17 years** **(*n* = 500)**	
**Child SDQ, mean (SD) **^**a**^						
Total difficulties score	11.9	(7.1)	12.1	(7.1)	11.7	(7.4)
Internalizing score	5.6	(3.8)	5.3	(3.7)	5.8	(4.0)
Externalizing score	6.3	(4.1)	6.8	(4.3)	5.8	(4.1)
**Parent PHQ-4, mean (SD) **^**b**^						
Total score	3.1	(3.3)	3.0	(3.3)	3.5	(3.4)
Anxiety score	1.6	(1.8)	1.6	(1.7)	1.8	(1.8)
Depression score	1.5	(1.7)	1.4	(1.7)	1.7	(1.8)
**Physical Activity**						
Child physical activity (days/week > 60 min), mean (SD)	3.9	(2.2)	4.1	(2.2)	3.4	(2.1)
Parent physical activity (MET-min/week), median (IQR)	3295	(6519)	3117	(6518)	3502	(6341)
Co-participation in outdoor time (days/week), mean (SD)	2.4	(2.2)	2.7	(2.2)	2.1	(2.2)

^**a**^The possible range of SDQ scores is 0–40 for the total difficulties score, 0–20 for the internalizing score, and 0–20 for the externalizing score. Twelve participants were missing data for one or more SDQ items and were excluded from the analysis

^**b**^PHQ-4 Total scores from 0–2 are considered normal, 3–5 are considered mild, 6–8 are considered moderate, and 9–12 are considered severe. Anxiety or depression scores can range from 0–6. Two participants were missing data for one or more PHQ-4 items and were excluded from the analysis

Children attained physical activity recommendations (at least 60 min of physical activity that raises the heart rate or breathing rate) on average 3.9 (SD 2.2) days per week (Table 2). Co-participation of parent and child in any outdoor time was reported for on average 2.4 days per week (SD 2.2). Spearman correlations between child PA and co-participation in outdoor time, and between parent PA and co-participation in outdoor time, were 0.34 and 0.43, respectively.

### Park access and mental health

Results from primary models of child behavior and parent mental health in the overall sample are shown in Table 3. Park access was associated with a lower SDQ total score among children (β -1.26, 95% CI: -2.25, -0.27), as well as with lower internalizing scores (β -0.67, 95% CI: -1.20, -0.13). Associations with externalizing scores were in the hypothesized direction but with confidence intervals that included the null. Park access was associated with a lower PHQ-4 total score among parents (β -0.89, 95% CI: -1.39, -0.40).

**Table 3:** Associations between park access and mental health during the COVID19 pandemic, in the overall sample and in stratified models by child age group

Outcome	Overall			Ages 6–10			Ages 11–17		
	**β**	**(95% CI)**	***p*-value**	**β**	**(95% CI)**	***p*-value**	**β**	**(95% CI)**	***p*-value**
**Child SDQ **^**a**^									
Total difficulties score	-1.26	(-2.25, -0.27)	0.013	-0.54	(-1.77, 0.70)	0.387	-1.91	(-3.23, -0.58)	0.014
Internalizing score	-0.67	(-1.20, -0.13)	0.014	-0.01	(-0.68, 0.66)	0.965	-1.04	(-1.74, -0.33)	0.010
Externalizing score	-0.56	(-1.16, 0.03)	0.062	-0.49	(-1.24, 0.26)	0.215	-0.88	(-1.66, 0.01)	0.052
**Parent PHQ-4 **^**b**^									
Total score	-0.89	(-1.39, -0.40)	< 0.001	-0.73	(-1.34, -0.11)	0.032	-0.24	(-0.85, 0.37)	0.551
Anxiety score	-0.39	(-0.65, -0.12)	0.005	-0.27	(-0.60, 0.06)	0.128	-0.15	(-0.49, 0.19)	0.478
Depression score	-0.50	(-0.77, -0.24)	< 0.001	-0.46	(-0.78, -0.14)	0.010	-0.08	(-0.41, 0.24)	0.691

^a^Models of child SDQ included adjustment for child age, gender, prior anxiety/depression, prior ADHD or behavioral problem, race/ethnicity, parent education, home ownership, school status, COVID-19 impact scale, and urbanicity

^b^Models of parent mental health included adjustment for parent gender, race/ethnicity, born outside the US, education, home ownership, COVID-19 impact scale, and urbanicity

In secondary analyses, models were fully stratified by child age group (6–10 versus 11–17 years). Statistically significant associations between park access and child SDQ scores were observed in the adolescent age group (Table 3). No associations with child mental health were observed among children ages 6–10 years old. Park access was associated with lower total and depression scores in parents of children ages 6–10, but not in parents of youth ages 11–17.

### Park access and physical activity

Results for linear regression analyses of physical activity are shown in Table 4. In adjusted models, estimates of the association between park access and child physical activity was in the hypothesized direction but confidence intervals included the null (β: 0.31 days of meeting PA recommendations, 95% CI: -0.03, 0.66). Park access was associated with higher levels of parent physical activity (β: 1009 MET-minutes/week, 95% CI: 301, 1717). This relationship was observed for parents of youth ages 11–17, but not for parents of younger children, in stratified models. Park access was also associated with 0.50 more days of co-participation of parents and their child in outdoor activity in the prior week (95% CI: 0.16, 0.84).

**Table 4:** Park access and physical activity during the COVID19 pandemic

Outcome	Overall			Ages 6–10			Ages 11–17		
	**β**	**(95% CI)**	***p*-value**	**β**	**(95% CI)**	***p*-value**	**β**	**(95% CI)**	***p*-value**
Child PA ^a^	0.31	(-0.03, 0.66)	0.078	0.17	(-0.28, 0.63)	0.455	0.15	(-0.34, 0.63)	0.553
Parent PA ^b^	1009	(301, 1717)	0.005	286	(-632, 1205)	0.541	1842	(678, 3006)	0.002
Co-participation in outdoor activity ^c^	0.50	(0.16, 0.84)	0.004	0.39	(-0.06, 0.84)	0.092	0.60	(0.10, 1.10)	0.018

^a^Models adjusted for child age, gender, school status, COVID-19 impact scale, parent education, home ownership, and urbanicity

^b^Models adjusted for parent gender, COVID-19 impact scale, parent education, race, born outside the US, home ownership, and urbanicity

^c^Models adjusted for parent gender, child gender, school status, COVID-19 impact scale, parent education, parent race, parent born outside the US, and urbanicity

### Extended models

In extended models, adjusting for co-participation or physical activity tended to attenuate the observed associations between park access and child SDQ scores (Table 5). However, adjustment for co-participation or physical activity did not meaningfully change the estimates of associations between park access and parent mental health.

**Table 5:** Sensitivity analysis adjusting for co-participation in outdoor time or physical activity in models of park access and mental health

Outcome	Adjusting for co-participation			Adjusting for PA		
	**β**	**(95% CI)**	***p*-value**	**β**	**(95% CI)**	***p*-value**
**Child SDQ **^**a**^						
Total difficulties score	-1.12	(-2.13, -0.11)	0.030	-1.04	(-2.04, -0.05)	0.039
Internalizing score	-0.63	(-1.17, -0.08)	0.024	-0.57	(-1.11, -0.04)	0.037
Externalizing score	-0.46	(-1.06, 0.13)	0.129	-0.44	(-1.03, 0.14)	0.139
**Parent PHQ-4 **^**b**^						
Total score	-0.85	(-1.34, -0.36)	0.001	-0.90	(-1.39, -0.41)	< 0.001
Anxiety score	-0.36	(-0.63, -0.10)	0.007	-0.38	(-0.65, -0.12)	0.004
Depression score	-0.49	(-0.75, -0.22)	0.000	-0.51	(-0.78, -0.25)	< 0.001

^a^Models of child SDQ also included adjustment for child age, gender, prior anxiety/depression, prior ADHD or behavioral problem, race/ethnicity, parent education, home ownership, school status, COVID-19 impact scale, and urbanicity

^b^Models of parent mental health also included adjustment for parent gender, race/ethnicity, born outside the US, education, home ownership, COVID-19 impact scale, and urbanicity

## Discussion

In this study, park access was associated with lower overall difficulties scores and fewer internalizing symptoms (emotional and peer problems) among children. Park access was also associated with more days of parent–child co-participation in outdoor time and with more parent physical activity. In this national U.S. sample, we found that perceived access to a park within a 10-minute walk from home was associated with better mental health for children and their parents during the first year of the COVID-19 pandemic.

The context of the COVID-19 pandemic, including major changes in school and work schedules and restricted access to indoor locations, provided a valuable opportunity to further understand the role of park access at residential locations for health. International studies have found that the COVID-19 pandemic has contributed to higher rates of anxiety, depression, and stress [27, 28]. Such population scale events highlight the need for community-level resources to address mental health during times of heightened stress. Studies from around the world indicate that visits to greenspace have increased during the pandemic [29–31]. This could be due to closure of other locations due to pandemic restrictions or self-awareness of the benefits of park visitation [32]. Importantly, during the pandemic residential greenness has been found to be associated with improved well-being and self-reported happiness, and fewer symptoms of depression and anxiety, [33–35]. The cross-sectional results from our study further support these findings.

Few studies have investigated relationships between park access and mental health across different age groups of children. A study of Canadian youth found that for those of age 11–17, access to parks in high density neighborhoods increased odds of outdoor activities during the pandemic [16]. Our study similarly identified statistically significant associations with mental health outcomes among youth ages 11–17. Taken together, these studies suggest that the importance of park proximity for both physical and mental health among adolescents. In both the Canadian study and in our U.S. study, associations between park access and mental health were not statistically significant when the sample was restricted to those ages 5–11 years. Mitra et al. hypothesize that younger children may have been more impacted by closure of school grounds and playgrounds. Prior to pandemic-related lockdowns, a study of adolescents ages 10–18 found that time spent in greenspace was associated with social contacts [36]. Interestingly, one study found variation in 12 year old’s anxiety levels based on distance to greenspace [37]. Our study builds on this prior literature highlighting the potential benefit of parks for adolescent health.

### Benefits pathways

Greenspaces, including parks, are theorized to improve mental health by promoting physical activity and social cohesion, or through direct effects on psychological or cognitive processing, including working memory, and physiological responses, including the stress response [12]. Extensive prior literature has focused on physical activity, finding the strongest associations between parks and physical activity using self-reported measures of neighborhood parks [38]. Studies have also suggested that physical activity mediates relationships between time spent outdoors and mental health [11]. Furthermore, physical activity in natural environments may be more beneficial for mental health than exercise indoors [39]. Access to outdoor spaces to exercise may be particularly important when access to other spaces for physical activity are limited. A sample of people across all ages in Hong Kong compared physical activity before and during the pandemic, and found that those in greener neighborhoods had a smaller decrease in physical activity than those in less green neighborhoods [40]. We were able to examine associations between park access and several intermediate outcomes, including both child and parent physical activity. However, associations with child physical activity, though in the hypothesized direction, were not statistically significant in our analysis. Programming within parks has also been shown to influence levels of physical activity [41] and the null results observed in our study may be due in part to the cancellation of organized activities during the pandemic. It is also possible that these associations were attenuated due to the late autumn timing of our survey, when people in many parts of the country are choosing not to engage in outdoor physical activities.

A novel contribution of this study was our examination of parent–child co-participation in outdoor time as an intermediate outcome. During the pandemic, social support and parent–child discussions have been identified as protective factors for mental health [27]. Specific patterns of parent attachment have previously been found to be associated with the development of internalizing problems in early childhood, which are in turn linked to later anxiety or depression [42]. Parents who have co-participated in physical activity with young children describe benefits for their children, including spending quality time together, improving children’s general health and well-being and the development of physical skills [43]. Qualitative studies have yielded parents’ recommendations to facilitate co-participation including home outdoor spaces, neighborhood design and play spaces, but to our knowledge this is the first quantitative study to examine the relationship between park access and co-participation of parent and child in outdoor activity. Our results indicate that park access may support families in spending time together outdoors, which in turn may facilitate such stronger bonds and social support. Further work is needed to test this potential pathway, including use of objective and/or validated outcome measures of co-participation and formal mediation analyses. In our study, parents reported the amount of co-participation in outdoor time with their child over the prior week, but this question has not been previously validated for use in research and thus these results should be interpreted cautiously. Future studies might include daily activity assessments to reduce recall bias or use of objective GPS and actigraphy measures to avoid the limitations of self-reported data, as well as using these methods to validate the self-report question used in this study. Co-participation in outdoor activity and nature experiences is an underexplored behavioral pathway to understanding factors related to outdoor physical activity and mental health status early in the human lifecycle. Our results suggest value in further investigations to promote health in young people.

The mental health benefits of park access observed in our study were sustained even when accounting for co-participation in time outdoors or overall physical activity, suggesting a potential role of other mechanisms. Theories of attention restoration and stress reduction also offer insights concerning some of the observed relationships between park access and mental health [44–46]. These psychophysiological theories, partially premised on psychoevolutionary perspectives, suggest that contact with nature environments directly facilitates restoration of cognitive resources and reduced activation of the physiological stress response. These underlying processes may in turn lead to improved mental health for both children and adults, and contribute to more positive child development [47].

### Implications for communities and planning

A social ecological framework posits that adaptations of policy, community, organizations, and social factors can generate changes in health behavior and health status [48], and that planning built environments to promote access to parks through safe, activity-friendly routes is a nationally recognized evidenced-based strategy to increase physical activity and promote health [49]. Our use of a micro-scale social ecological framework captured multiple influencing factors, considering parks as community conditions, and co-participation as social and interpersonal conditions that may influence health status. Our study and related findings have implications beyond the pandemic, suggesting that if communities plan and design urban greenspaces systematically for health and wellbeing, such park investments can generate numerous beneficial health outcomes [50–53] and potentially provide return on investment [54]. Urban parks and greenspace can provide residents a nature-based resource during a pandemic or other challenges (such as heat episodes) to maintain favorable health and quality of life [55]. Community-level program and policy interventions to increase safe access to parks may improve mental health and well-being among children and parents as well as bolster resilience during times of heightened stress [56].

Our study outcomes have implications for more precision oriented parks planning, that may even extend to tactical urbanism to meet health goals [57]. For instance, neighborhood characteristics influence park use, as one study found that access to parks in high-density neighborhoods was associated with increased participation in outdoor activities by youth during the pandemic [16]. The age of park users may also inform planning. Greening schoolyards is an intervention to transform playgrounds into community parks, but this approach has primarily targeted elementary schools. Prior studies indicate improved academic performance and stress response in response to nearby nature on high school campuses [58, 59]. If schoolyard renovations that transform those spaces into community parks also target middle and high schools where youth spend much of their time, these spaces may be an important health resources for adolescents.

Underlying all parks initiatives is the need to address equity [18, 60]. An estimated 100 million people in the U.S. do not currently have access to parks within a 10-minute walk, including 28 million children [18]. Analyses have revealed disparities in park and greenspace availability by race/ethnicity and socioeconomic status across many cities, particularly when examining park acreage and park quality, with neighborhoods with lower socioeconomic status and a higher percentage of people of color having less access to large high-quality parks [61]. Disparities in access to recreational facilities have been linked to reduced physical activity and overweight patterns in U.S. adolescents [62]. Furthermore, park quality and amenities may also not be equitably available, influencing children’s satisfaction with parks [63]. There are likely additional barriers for some families to engage in park-based health behaviors based on factors such as socioeconomic status, parents’ job status (e.g., able to work from home, “frontline” worker, or unemployed), and neighborhood conditions. Intervention opportunities such as transforming schoolyards to parks through land use agreements, rails to trails projects, and vacant lot conversions can be prime opportunities to increase access within regions with limited lands for acquisition.

### Strengths and limitations

The strengths of this study include the sampling and weighting of respondents in this study to be nationally representative, improving generalizability. The validated tools used to assess mental health outcomes provided a condition array and continuous measure of symptoms. We were also able to account for some potentially important confounders specific to the time at which the survey was administered, including school status and the COVID-19 family impact scale. Revised lifestyles during this time due to widespread lockdowns were also an opportunity to test for a family level social dynamic that has implications for child psycho-social health. An important contribution of this study to the literature on greenspace and mental health is the focus on the family unit.

There were also several limitations to acknowledge in this study. The cross-sectional structure of this dataset limits causal inference. Residual confounding by socioeconomic factors may explain the observed correlations between park access and mental health, a general limitation of much of the greenspace literature, despite the inclusion of several potential confounders in this analysis. We were unable to compare health outcomes before and after pandemic restrictions or to conduct a formal mediation analysis. All exposures, confounders, and outcomes were reported by participants and may be subject to same-source, recall, and social desirability biases, potentially resulting in systematic under- or over-reporting leading to biased estimates. Similar to many other studies of park proximity and health, we do not know if or how much time respondents actually spent in the park that they reported living close to or the type of activity they participated in during their visits. In addition, conditions of safety were reliant on respondents’ perception, suggesting an additional nuance to explore in future research. The survey question regarding park access did not differentiate between nature-based parks and parks with limited greenspace or health promoting design features and amenities. Further research on nature-related characteristics of the park, length of engagement, vigorousness of activity, characteristics of co-participation, types of activities, seasonal differences, and the spaces in which activities were carried out can inform both programming to promote healthy socialization as well as the design of parks and the built environment.

## Conclusions

Our cross-sectional study identified associations between reporting access to a park within a 10-minute walk with fewer reported parent and child mental health symptoms. Additionally, this study found an association between park access and participation of parents and children in time spent outdoors together. In the context of high-stress long-term events of the COVID-19 pandemic, opportunistic changes may create unique pathways to buttress against deteriorating mental health and well-being status. Our novel finding in secondary analyses of associations between park access and co-participation in outdoor time is an understudied pathway within research work on greenspace and health that should be formally investigated as a mechanism in future work.

Though this study examined the relationship between park access and mental health specifically in the context of the first year of the COVID-19 pandemic, insights from this work contribute to a broader body of literature that potentially informs our understanding of community resources and health during the pandemic moving forward, as well as during future large-scale events of heightened psychophysiological stress. Consideration of pathways at multiple microscales, including individual, family, and community levels can inform our understanding of relationships between park access and health outcomes. Neighborhood design and community programming interventions beyond the COVID-19 pandemic, particularly interventions for youth, may serve as a resource in increasing physical activity and supporting age-related trajectories of improved health. While the built environment, specifically park access, may require long-term investment and development processes, interim efforts to increase opportunity to do outdoor or nature-based activities within households and across generations may promote healthy behaviors and improve health status.

## Data Availability

The datasets used and/or analyzed during the current study are available from the corresponding author upon request.

## Abbreviations

SDQ: The Strengths and Difficulties Questionnaire
PHQ-4: The Patient Health Questionnaire-4
PA: Physical activity
CI: Confidence Interval
SD: Standard Deviation
MET: Metabolic Equivalent of Task
